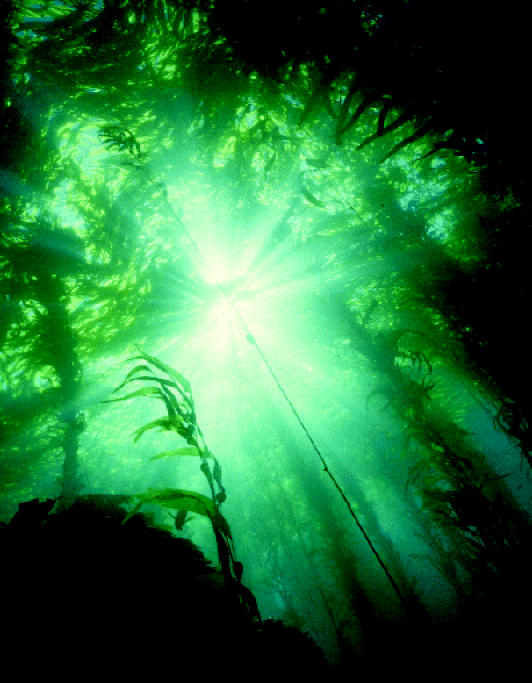# The Beat

**Published:** 2004-11

**Authors:** Erin E. Dooley

## Jakarta’s New Monorail

Jakarta has begun a monorail project to combat the air pollution and massive traffic jams that plague the city of nearly 9 million people and 5 million vehicles. The monorail system, still in the planning phases, is expected to consist of two lines, one serving the Indonesian capital’s central business district and the other running several miles through the city’s outer areas. The city has also reserved two lanes of the city’s main thoroughfare for bus traffic only.

Jakarta’s air quality is ranked among the worst in the world. Studies have found that the sixth leading cause of death in Indonesia is inflammation of the respiratory tract, which is closely linked with poor air quality.

## U.S. Climate Changes

In August 2004, the Bush administration delivered a report to Congress acknowledging that emissions of greenhouse gases are the best explanation for the global warming trend of the past 30 years. The report, which is available on the Internet at **http://www.climatescience.gov/**, was prepared by the U.S. Climate Change Science Program and the Subcommittee on Global Change Research, which are made up of federal representatives. The report also cited specific risks to farmers: increased carbon dioxide emissions stimulate the growth of invasive weeds, while reducing the nutritional value of certain grasses. Some industry groups dispute the new report, stating the science behind it is flawed.

## LA’s Shipshape Terminal

The Port of Los Angeles is home to the world’s first container terminal using Alternative Maritime Power (AMP) technology. Although this technology has been used by the U.S. Navy since World War II, the shipping industry has resisted adopting it due to cost and time issues. AMP technology provides a dock with electricity that is converted to a ship-compatible voltage. Ships plug in to the dock instead of running their diesel engines during loading and unloading, cutting emissions of smog-forming nitrogen oxides by 1 ton and particulate matter by 87 pounds per day.

China Shipping Lines, which leases the new berth, has agreed to retrofit 11 of its ships for the new power source. The first vessel plugged in to the updated berth in June 2004.

## Rain Theft in China

With some agricultural areas of China in the grip of an extended drought, cities have turned to rain-making technology to extract precious water from the skies. Now neighboring cities in Henan province are accusing one another of an unusual crime: rain theft. In July 2004, Zhoukou city officials claimed that rain makers in Pingdingshan overseeded clouds so that the latter city enjoyed rainfall that should have been Zhoukou’s. City officials want the courts to set up laws for “cloud farming,” although scientists believe the technology is not yet proven enough to regulate.

China is one of the world’s leading users of rain-making technology, which involves seeding cumulus clouds with dry ice or silver iodide to prompt precipitation. The Chinese government has set aside approximately US$50 million for nationwide weather management systems. Many local and provincial governments have set up “weather modification” bureaus charged with cloud seeding.

## AHA Links Pollution to Heart Disease

In the 1 June 2004 issue of *Circulation*, the American Heart Association made its first firm policy statement linking heart disease and long-term exposure to air pollution. The statement, written by University of Michigan researchers, is based on an extensive literature review. It cites particulate matter such as that generated by traffic as especially dangerous. The statement also points to a clear association between secondhand tobacco smoke and heart disease. Lead author Robert Brook called the link between air pollution and heart disease “a serious public health problem” because of the large number of people affected and because exposure occurs over a lifetime.

## Seaweed Attacks DDT

An international research team funded by the Royal Thai government has found that applying powdered seaweed to soil contaminated with the pesticide DDT can accelerate the breakdown of the contaminant. DDT was widely used from its introduction in the 1940s until it was banned in the United States in 1972. It is still used for mosquito control in some countries where malaria is prevalent. The researchers, whose work appeared in the June 2004 issue of the *Journal of Chemical Technology and Biotechnology*, found that the optimal proportion of 0.5% seaweed by weight resulted in 80% of the DDT degrading within six weeks. The sodium in the seaweed loosens the soil, allowing microorganisms to reach and attack the DDT.

## Figures and Tables

**Figure:**
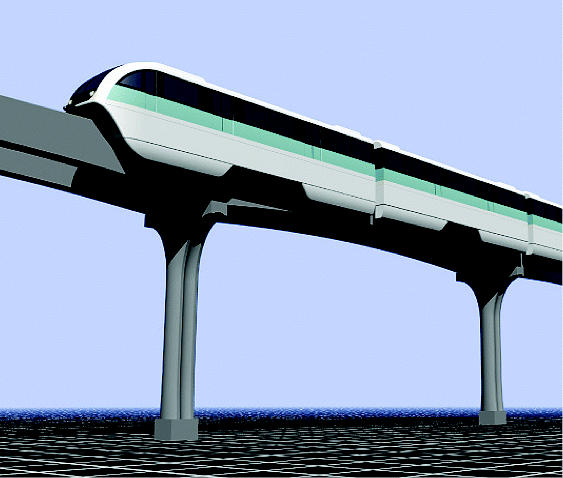


**Figure:**
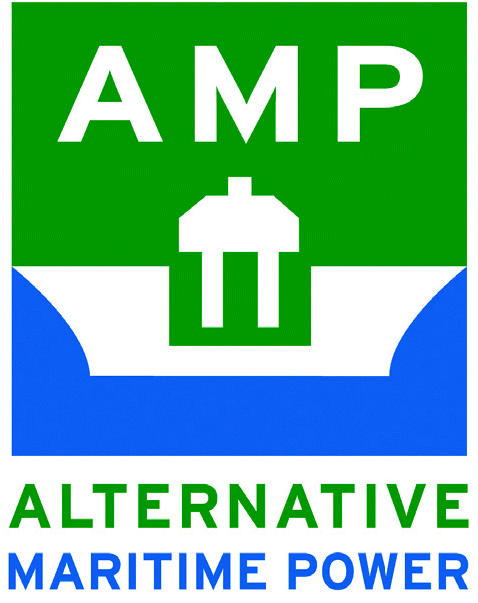


**Figure:**
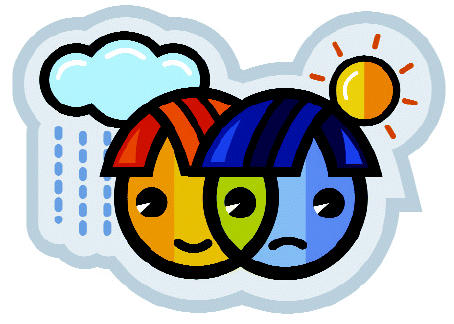


**Figure:**